# Examining the validity of the polish short form version of the self-regulated learning—sport practice survey among competitive athletes

**DOI:** 10.3389/fpsyg.2023.1132608

**Published:** 2023-02-02

**Authors:** Malgorzata Siekanska, Stuart G. Wilson, Jan Blecharz, Bradley W. Young

**Affiliations:** ^1^Department of Psychology, University of Physical Education in Krakow, Krakow, Poland; ^2^School of Human Kinetics, University of Ottawa, Ottawa, ON, Canada

**Keywords:** self-regulation, sports level, expertise, self-assessment, metacognition, motivation regulation

## Abstract

**Introduction:**

Self-regulated learning entails psychological processes that elite athletes employ to optimize their practice. Although self-regulated learning provides insights into athlete-led practice, research has been limited to few cultures, and the particularities of how SRL surveys perform in new cultural contexts require attention. Moreover, there exists no measure to assess SRL and its relationship to quality sport practice in Polish. Thus, we examined the Short Form of the Self-Regulated Learning**—**Sport Practice survey in Polish. Analyses addressed the factorial validity and reliability, the criterion validity (by assessing differences in scores between competition levels), and the concurrent validity (by correlating scores with conceptually related constructs) of a Polish Short Form survey.

**Methods:**

Athletes (*N* = 324, *M*_age_ = 21.4, *n*_females_ = 144, *n*_males_ = 180) from amateur, regional, national, and international-elite levels completed the survey, along with concurrent subscales (General Self-Efficacy Scale; GSES; Metacognitive-Self Scale; MS-24; Action Control Scale; ACS-90).

**Results:**

Confirmatory factor analysis indicated a two-factor (metacognitive; motivational) model (RMSEA = 0.082, SRMR = 0.057, CFI = 0.89). Between-group tests showed international-elite scoring higher than all other groups on metacognitive and motivational subscales. On both subscales, significant trends indicated that more skilled levels consistently reported higher scores than lesser-skilled levels. The short form scores were associated with certain concurrent variables, including GSES (*r_meta_* = 0.41, *r_motiv_* = 0.48), MS-24 (*r_meta_* = 0.39, *r_motiv = 0_*.24), and ACS-90 (AOF subscale: *r_motiv = 0_*.26).

**Discussion:**

On the basis of strong criterion validity, and moderate evidence for concurrent validity, we conclude that the Polish Short Form of the Self-Regulated Learning**—**Sport Practice survey is a promising tool for use in Polish sport and we discuss future avenues of work to enhance its validation. Limitations that inform future research include our reliance on a mixed-sport sample, the lack of priming of obstacles/challenge ahead of self-report, and a lack of consideration of sport-specific practice variables in analyses.

## Introduction

Self-regulated learning (SRL) has received a lot of recent attention in the sport context (Toering et al., 2012a; McCardle et al., 2018; Wilson et al., 2021). Evidence suggests that SRL, which comprises active, self-directive psychological processes, is useful for improving practice activities because it can help learners to self-monitor, regulate, and control cognition, motivation, affect, behavior, and aspects of the environment to achieve learning goals (Kolovelonis et al., 2012). SRL competencies are related to effective sport practice and to the achievement of higher skill levels (Anshel and Porter, 1996; Bartulovic et al., 2017), which suggests that self-regulated athletes get more out of their athletic potential (McCardle et al., 2017). An instrument that effectively measures SRL may help to identify athletes’ strengths and weaknesses with respect to optimal psychological engagement in practice activity (Young et al., in press).

### Self-regulated learning research across different sport cultures

Self-report measures of SRL have been investigated in a limited selection of cultural contexts. Toering et al. (2012a), based in the Netherlands, created the first self-report survey for SRL in sport by combining existing scales from education research in English. They translated the survey to Dutch and assessed factorial validity for their 48-item, six subscale model in a sample of Dutch youth academy soccer players, advancing four metacognitive subscales: “planning,” “self-monitoring,” “self-reflection,” and “self-evaluation,” and two motivational subscales: “effort” and “self-efficacy.” Subsequent studies found that higher-performing Dutch adolescent athletes scored higher than less-elite counterparts across multiple sports (e.g., Jonker et al., 2010), and more specifically, that SRL scores were related to ball control development among basketball players (te Wierike et al., 2018).

English versions of Toering et al. (2012a) survey have been extensively examined and updated for use in a North American (primarily Canadian) context. Bartulovic et al. (2017) worked with domain experts to adapt the survey to the context of sport practice and found that their survey effectively discriminated between three performance levels in a mixed-sport sample. McCardle et al. (2018) sought to reinforce the conceptual and psychometric validity of Bartulovic et al.’s survey through extensive psychometric analyses to refine and validate an expanded inventory of items. Their work resulted in 26 items in the Self-Regulated Learning for Sport Practice (SRL-SP) survey. Their five-subscale solution differed from Toering et al. (2012a) six-factor one by integrating self-reflection and self-evaluation, changing “self-monitoring” to “checking,” and noting that self-efficacy was assessed in relation to challenges. Recently, Wilson et al. (2021) replicated the five-subscale structure and its factorial validity in another sample of North American athletes. They also found that criterion validity (i.e., differences between four skill-level groups) was retained after controlling for the athletes’ biases toward social desirability.

Other investigators have adapted versions of the preceding surveys to new cultural contexts. Pitkethly and Lau (2016) translated the Toering et al. (2012a) survey to Chinese and validated a model with 32 items on six adjusted subscales among two samples of adolescent (non-athlete) Hong Kong students. Ikudome et al. (2017) translated the survey to Japanese, and validated a five-subscale, 37-item solution among university students within school sport clubs. Reverberi et al. (2021) translated Bartulovic et al. (2017) survey to Italian, finding a five-subscale solution (31 items) that showed measurement invariance between male professional and semi-professional footballers in Italy. Peer-reviewed self-report measures of SRL now exist in North American (English), European (Dutch, Italian), and East Asian (Chinese, Japanese) contexts.

Each time an SRL survey has been translated to a new language and/or cultural context has necessitated a slight adjustment to the survey factor structure (5–6 factors) and item selection (26–48 items). Some changes are attributable to minor linguistic modifications. For instance, Bartulovic et al. (2017) changed the word “problem,” representing the school-related origin of the scale, to “task” to better represent sport practice. Pitkethly and Lau (2016) used “work” instead because it was more easily understood in translation to Chinese. They also noted that cultural differences in motivational beliefs may affect inter-scale correlations, such as whether effort is invested more for personal gain (i.e., as in more individualist societies) or for the benefit of the group (i.e., more collectivist societies). Although SRL assessment has gained popularity in different countries, it is not known whether the construct is received similarly by respondents, and whether it performs equally in terms of criterion validity, in different cultures. Thus, the current investigation explored the performance of an SRL survey tool in a new cultural context—among Polish competitive athletes.

Despite the various self-report SRL tools, the shortest validated surveys have used 26 to 31 items, which may be too long for use in applied sport consultation settings (Horvath and Röthlin, 2018). In response, Wilson et al. (2019) developed a Short Form of the SRL-SP through secondary analysis of McCardle et al. (2018). A panel of five researchers holding different areas of SRL expertise (e.g., applied consulting, psychometric, theoretical) re-appraised McCardle et al.’s inventory of items and selected 14 for inclusion based on a combination of conceptual and practical merit. Exploratory factor analysis of 482 North American athletes (*M*age = 26.45, *SD* = 12.66) indicated a two-factor solution—motivation and metacognition subscales—and skill group comparisons among athletes (> 17 years of age), indicating that international athletes scored correspondingly higher than national and provincial athletes on both subscales. Although Wilson et al. presented their Short Form SRL-SP as a viable tool for assessing self-regulated sport practice in applied North American settings with English-speaking athletes, it has yet to be examined in alternative cultural contexts. The current investigation sought to examine this Short Form SRL-SP among Polish athletes.

### Criterion validity based on between-group comparisons

Sport expertise researchers have contended that a survey should establish criterion validity by discriminating between multiple, escalating skill groups in a corresponding manner (i.e., higher scores represent higher skill levels) with noted effect sizes (Tedesqui et al., 2018; Wilson et al., 2021). For example, researchers have consistently demonstrated that higher-performing football (soccer) players in the Dutch youth football academy system score higher than their non-elite peers on the SRL subscale of “self-reflection” (Toering et al., 2009, 2012b; Jonker et al., 2010, 2012). Bartulovic et al. (2017) found that subscales of “planning,” “self-monitoring,” “effort,” and “self-efficacy” each predicted membership in the elite group of a North American mixed-sport sample, as compared to less-elite and recreationally competitive groups, but that only “self-monitoring” did so when considered simultaneously with all other subscale scores. McCardle et al. (2018) and Wilson et al. (2021) both found that international-level athletes from a North American mixed-sport sample scored significantly higher than lesser-skilled athletes on processes of “evaluation/reflection” and “effort.” McCardle et al. also found significant differences for “self-efficacy for challenge,” although Wilson et al.’s findings also implicated more subscales attesting to criterion validity than McCardle et al. (2018) as trends relating to “planning, “checking,” and ‘self-efficacy for challenge’ subscale scores all pointed to an expert advantage. Finally, Reverberi et al. (2021) found that professional Italian male football players consistently scored higher than semi-professional players across all five subscales. They found small between-group differences for “planning,” “self-reflection,” and “self-efficacy” scores, medium differences for their “self-supervision” factor, and strong differences on the ‘effort’ factor.

When researchers neglect to examine inter-group skill differences (e.g., Pitkethly and Lau, 2016; Ikudome et al., 2017), this curtails research practitioners from fully interpreting the validity of the instrument. Although each survey that tested inter-group differences did demonstrate criterion validity, the varying importance of different subscales (e.g., “self-reflection,” “effort”) between samples suggests that cultural differences in how SRL processes are perceived.

### Concurrent validity

Despite extensive investigation of various SRL surveys, little work has assessed how the results of these measures correspond to those of similar constructs (*cf.*, Elferink-Gemser et al., 2015). SRL embraces motivational self-processes (e.g., self-efficacy) and metacognitive capabilities (e.g., planning and self-monitoring) focused on practice-enhancement. Many of these elements can be similarly measured using corresponding single-dimension (e.g., General Self-Efficacy Scale, Schwarzer et al., 2009; Metacognitive-Self Scale, Brycz and Konarski, 2016) or multidimensional scales (e.g., Action Control Scale, Kuhl, 1994a; Marszał-Wiśniewska, 2002). Although these corresponding measures are more general than sport-specific, they have been used in athlete research, in Polish (e.g., Marszał-Wiśniewska, 1998; Blecharz et al., 2014; Wilczynska et al., 2014; Serafin, 2021; Rogowska et al., 2022) and other populations (e.g., Raab and Johnson, 2004). Self-efficacy, for example, is critical to self-regulation and is understood as modulating one’s behavior to achieve goals (Rogowska et al., 2022). Basketball experts report higher self-efficacy than non-experts and novices in a practice environment demanding SRL (Cleary and Zimmermann, 2001).

There are parallels between SRL and facets of the metacognitive self. For example, Serafin (2021) reported that kickboxers achieved metacognitive self (MCS) levels above the average for the general population, suggesting they possess well-developed insight regarding their own evaluative biases in action, can accurately predict their own behavior, and have capabilities that allow for conscious correction of mistakes. Research has shown that high MCS fosters self-regulatory functions such as persistence in the face of challenge and focus on non-conflicting goals (Fanslau and Brycz, 2019). Moreover, high-MCS individuals accept uncontrollability more than low-MCS ones (Brycz et al., 2014).

According to action-control theory, volitional capacities are required to translate goals into behavior and are essential for the initiation of action and monitoring goal-relevant behavior in the face of obstacles (Kuhl, 1994b). With respect to such self-regulation, Kuhl (1994a, 1994b) specified that people have either state- or action-oriented mechanisms. Action-oriented people are characterized by mobilization, high activity, and high efficiency of the internal mechanisms of self-regulation, so they can efficiently formulate an action plan as well as take such an action. State-oriented people have problems with planning and initiating activities, and ruminating on setbacks. Research on basketball players showed that state-oriented players regulate poorly and have weaker sporting achievements (Marszał-Wiśniewska, 1998). In light of the aforementioned parallels between SRL and related constructs, one would expect concurrent validity in a Polish athletic cohort to be reflected in associations between SRL scores and higher general self-efficacy, higher MCS scores, and action-control mechanisms.

### Research purposes

Although the catalog of SRL surveys is being used more widely, research practitioners cannot assume any one of them, including the Short Form SRL-SP, validly applies across cultures without understanding its validity within a specific cultural context. Validity in a sport performance context should be established through the intersection of multiple considerations, including factorial, criterion, and concurrent validity. As such, this investigation specifically examined the Polish version of the Short Form SRL-SP survey’s psychometric characteristics and its constituent themes in a Polish sample, tested the criterion validity of the survey scores *via* inter-group assessment of Polish athletes of different sport levels, and examined its concurrent validity in relation to parallel scores for general self-efficacy, metacognitive self, and action control.

## Methods

### Participants

Participants were recruited *via* organizational and coach contacts at sports university, clubs, and institutions, and were approached onsite at events or practices around Poland (mainly in Lesser Poland). Participants had to be 18 years of age or older, and actively practicing for and competing in organized sport at either amateur, regional, national, or international-elite levels. We recruited 324 Polish athletes (*M*_age_ = 21.4, *n*_females_ = 144, *n*_males_ = 180) from diverse sports (individual, *n* = 161, and team sports, *n* = 163). To improve the validity of skill grouping (Tedesqui et al., 2018), sport levels were determined based on three questions in a demographic survey. The first asked for the official sport-level classification (i.e., international master class, master class, first, second, third class, etc.) used by particular Polish sport associations. The rules for assigning sport class are based on official state regulations, and a sports class is valid for a maximum of 2 years. The second question asked athletes to report their competitive level (i.e., amateur; regional, national, elite), and the third requested the athlete’s greatest achievement in their sport. Based on these multiple criteria, we classified respondents into four escalating sports levels: amateur, *n* = 55; regional, *n* = 116; national, selected to represent Poland, *n* = 117; international elite, *n* = 36.

### Procedures

The research was carried out in accordance with the Helsinki Declaration and ethics procedures that received approval from an academic institution. During an introductory meeting, participants were introduced to the idea of the study and ethical considerations, including informed consent. Surveys were disseminated either in group or in individual sessions, with completion taking about 35 min. Participants completed a demographic survey and Polish versions of the Short Form SRL-SP survey (back-translated from Wilson et al., 2019), the General Self-Efficacy Scale (GSES; Schwarzer et al., 2009), the Metacognitive-Self Scale (MCS-24; Brycz and Konarski, 2016), and the Action Control Scale (ACS-90; Kuhl, 1994a). The latter three surveys already existed in Polish.

### Measures

#### Polish short form SRL-SP

The English Short Form SRL-SP consists of 14 items that assess an athlete’s use of metacognitive (10 items; α = 0.87) and motivational (four items; α = 0.73) self-regulated learning processes (Wilson et al., 2019). All items are assessed on a 7-point scale, where metacognitive items are anchored at each point from ‘never’ to ‘always’, and motivational items are anchored at each point from ‘strongly disagree’ to ‘strongly agree’ (see Appendix I). Permission to translate and use the Short Form SRL-SP was obtained from the original authors (Wilson et al., 2019). First, a native Polish speaker and certified sport psychologist translated this survey into Polish. Then, a professional translator who was not familiar with the SRL content performed a back-translation. Two discrepancies (items 2 and 6) between the two versions were found. After a discussion with the original authors, corrections were made. This version of the Short Form was next verified with a certified sport psychology practitioner to ensure it was faithful to the scope and the language of Polish athletes; only two words in the survey preface were replaced (Appendix I).

#### General self-efficacy scale

The GSES (Schwarzer et al., 2009) is a 10-item instrument that explicitly refers to the belief that one’s regulatory actions are responsible for successful outcomes (e.g., “I can always manage to solve difficult problems if I try hard enough”). All items are assessed on a 4-point scale anchored at 1—“Not at all true” and 4—“Exactly true.” Higher scores indicate a stronger self-efficacy belief. The internal consistency (Cronbach’s α) of the GSES ranges from 0.85 to 0.90 in Polish athletic samples (Łuszczyńska et al., 2005; Juczyński, 2009).

#### Metacognitive-self scale

The MCS-24 (Brycz and Konarski, 2016) is a 24-item scale that assesses knowledge about one’s own adaptive biases and about the influence of psychological phenomena on one’s own behavior (e.g., “I remember information better when I can relate it to the knowledge I already have”). Participants are asked to rate their agreement with each item on a scale anchored at 1—‘Definitely NO’ and 6—‘Definitely YES’ (Brycz and Konarski, 2016). Higher scores indicate a better understanding of how psychological mechanisms and metacognitive biases influence oneself, and better support for self-regulatory functions for behaviors in different areas of life activities (including sport). In a Polish sample, Brycz and Konarski (2016) reported that the discrimination parameters for all test items were statistically significant, ranging from 0.42 to 0.96, and internal consistency values were α = 0.81 and ω = 0.85.

#### Action control scale

The ACS-90 (Kuhl, 1994a; Marszał-Wiśniewska, 2002). assesses volitional capacities and mechanisms that facilitate (or impede) the enactment of intentions. This 36-item measure consists of a series of items that require respondents to choose between two alternatives. For example, after being prompted with “When I know I must finish something soon,” they choose: (a) “I have to push myself to get started” (i.e., hesitation orientation) or (b) “I find it easy to get it over and done with” (decision-related action orientation). Following “When I have to carry out an important but unpleasant task,” they choose: (a) “I do it and get it over with” (i.e., action orientation) or (b) “It can take a while before I can bring myself to do it” (state orientation; Kuhl, 1994a; Marszał-Wiśniewska, 2002). Action-oriented choices are coded as 1 and state-oriented choices as 0, and these are summed, for 12 items on each of three subscales. The three subscales are: (i) action orientation subsequent to failure vs. preoccupation (AOF); (ii) prospective and decision-related action orientation vs. hesitation (AOD); and (iii) action orientation during (successful) performance of activities (intrinsic orientation) vs. volatility (AOP). The subscales were treated and summed separately. Each of the resulting subscale scores was analyzed as continuous variables, with higher scores representing more action orientation.

### Planned analyses

We initially assessed for missing values. Three MCS-24 items contained one missing value representing <0.62% of data. These values were missing at random and were thus replaced using randomly generated values within the range of pertinent scales. We conducted confirmatory factor analysis (CFA), using obliminal rotation^1^ and maximal likelihood estimation, to evaluate the factorial structure and internal consistency of scores from the Polish Short Form SRL-SP. We performed as per the English version (Wilson et al., 2019), two CFAs, one for a global factor and a separate analysis for the two-factor model. Multiple criteria were used to assess fit, including root mean square error of approximation (RMSEA) < 0.05, standardized root-mean-square residual (SRMR) < 0.08, comparative fit index (CFI) > 0.90, and Tucker-Lewis Index (TLI) > 0.90 (Kenny, 2020). We assessed subscale reliability with Cronbach’s α, with values >0.70 considered acceptable (Nunnally, 1970). Additionally, we reported Average Variance Extracted analysis (AVE).

In terms of criterion validity, we assessed differences between the four competitive levels (amateur, regional, national, and international elite) using Kruskal-Wallis ANOVA and *post-hoc* Dunn tests. Effect sizes for ANOVAs were based on partial eta-squared values interpreted as 0.01 small, 0.06 medium, and 0.14 large. Effect sizes for *post-hoc* comparisons were based on *d* values interpreted as.2 small, 0.5 medium, and.8 large (Cohen, 1988). Preliminary analyses assessed differences in SRL scores between genders. In terms of concurrent validity, after performing Shapiro–Wilk analyses for normality, we conducted Spearman’s rho correlations between each of the motivational and metacognitive Short Form SRL-SP subscale scores and the other notable self-regulation related variables (i.e., GSES, *n* = 291; MCS-24, *n* = 323, and the three scales of action control, *n* = 198^2^). All analyses were performed using JASP 0.16 software, with the significance level set at *α* < 0.05.

## Results

### Factorial validity and internal consistency reliability

The CFA on the global factor model showed the following fit indexes: RMSEA = 0.100 (CI 0.089–0.111), SRMR = 0.072, CFI = 0.829, and TLI = 0.798. The CFA for the two-factor model showed better fit. The items on the two factors matched the English Short Form SRL-SP (Wilson et al., 2019) exactly: ‘metacognitive’ (10 items, factor loadings 0.52–0.66) and ‘motivational’ (4 items, factor loadings.64–0.70) subscales, which were correlated at *r* = 0.68 (see Table 1). The two-factor model fit the data as follows: RMSEA = 0.082 (CI.070–0.093) SRMR = 0.057, CFI = 0.887, and TLI = 0.869. Standardized loading estimates should be 0.5 or higher, and ideally.7 or higher (Hair et al., 2019). In our study, Cronbach’s α for the metacognitive and motivational subscales were 0.85 and 0.77, respectively. In light of these results, and considering the two-factor model allows for more nuance in facets of SRL, we elected to use the metacognitive and motivational scores in our subsequent analyses.

**Table 1:** Mean, standard deviations, standard error, variance, and factor loadings based on the confirmatory factor analysis.

Item		*M*	*SD*	SE	Variance	Factor loadings	
						Metacognition	Motivation
1. I try to understand the goal of a practice task before I do it.		5.50	1.33	0.07	1.77	0.56	
2. I consciously have goals in mind for how hard I want to work at practice.		5.97	1.18	0.07	1.38	0.56	
3. I check how well I am doing during practice tasks.		5.75	1.13	0.06	1.28	0.52	
4. I clearly plan my course of action before starting practice tasks.		5.26	1.31	0.07	1.70	0.57	
5. During practice, I consciously have goals in mind to improve how I train.		5.86	1.16	0.06	1.34	0.59	
6. I reflect upon my actions at practice to see whether I can improve them.		5.82	1.17	0.06	1.37	0.64	
7. Before I do a practice task, I think through the steps in my mind.		5.37	1.32	0.07	1.75	0.61	
8. When thinking about my practice, I reflect about my strengths and weaknesses.		5.21	1.48	0.08	2.20	0.56	
9. I develop a plan for resolving difficulties at practice.		4.85	1.37	0.08	1.88	0.64	
10. After finishing, I look back on practice tasks to evaluate my performance.		4.74	1.56	0.09	2.44	0.66	
11. Even when I do not like a task during practice, I work hard.		5.69	1.20	0.07	1.45		0.68
12. When facing difficulties at practice I can rely on my coping abilities.		5.61	1.05	0.06	1.09		0.64
13. I am confident that I can deal efficiently with unexpected events at practice.		5.43	1.10	0.06	1.21		0.65
14. I usually keep working hard even when sport training tasks become difficult.		5.95	1.03	0.06	1.07		0.70
Factor characteristics							
Factor	*M* of Total Score (*SD*)	*M* of Average Score (*SD*)	AVE	Unrotated solution		Rotated solution	
				Sum Sq loadings	Proportion variance	Sum Sq loadings	Proportion variance
Metacognition	54.34 *(8.48)*	5.43 (0.85)*	0.35	4.82	0.35	3.14	0.22
Motivation	22.68 *(3.38)*	5.67 (0.84)*	0.45	0.88	0.06	2.57	0.18
Cumulative variance					0.41		0.41

Cronbach’s α for the metacognitive and motivational subscales were.85 and.77, respectively; AVE – Average Variance Extracted. Total scores are out of 70 for metacognition, out of 28 for motivation; *average score for each factor on a 1–7 scale.

### Criterion validity

Preliminary analyses indicated scores between female and male athletes did not differ (*p_meta_* = 0.06; *p_motiv_* = 0.58). A one-way Kruskal-Wallis ANOVA for metacognitive scores was significant, *H* (*n* = 3, 324) = 15.58, *p* = 0.001, partial eta-square = 0.05. Dunn tests showed significant differences between all of the levels with one exception—the regional athletes’ levels were not different from than amateurs (see Table 2). A one-way Kruskal-Wallis ANOVA for motivational scores was significant, *H* (*n =* 3, 324) = 23.22, *p* < 0.001, partial eta-square = 0.07. *Post-hoc* tests showed significant differences between all of the levels with one exception—the national group was not different from the regional group (see Table 2). Notably, inspection of descriptive statistics indicated “complete correspondence” (Tedesqui et al., 2018, p: 7), that is, with each increasing sport competition level, there was higher report of metacognitive and motivational scores.

**Table 2:** Inter-group data for scores on the metacognitive and motivational subscales of the Polish Short Form SRL-SP.

Factor	Competitive level	Mean total score / mean of average score (*SD*)*	*Post-hoc* Dunn test statistics			
			Between-group comparisons	*Z*	*p*	*D*
Metacognitive score	Amateurs	52.02 *(8.65)* / 5.20 *(0.86)*	Amateurs–Regional	−0.758	0.224	0.170
	Regional	53.45 *(8.04)* / 5.34 (0.80)	Amateurs–National	−2.282	0.011	0.360
	National	55.21 *(9.00)* / 5.52 *(0.90)*	Amateurs–International Elite	−3.442	< 0.001	0.758
	International Elite	57.89 *(6.33)* / 5.79 *(0.63)*	Regional - National	−1.900	0.029	0.211
			Regional–International Elite	−3.217	< 0.001	0.589
			National–International Elite	−1.914	0.028	0.319
Motivational score	Amateurs	21.13 *(3.38)* / 5.28 *(0.85)*	Amateurs–Regional	−2.517	0.006	0.370
	Regional	22.40 *(3.48)* / 5.60 *(0.87)*	Amateurs–National	−3.810	< 0.001	0.621
	National	23.21 (3.33) / 5.80 *(0.83)*	Amateurs–International Elite	−4.383	< 0.001	1.076
	International Elite	*24.25 (1.87)* / *6.06 (0.47)*	Regional–National	−1.609	0.054	0.235
			Regional–International Elite	−2.769	0.003	0.576
			National–International Elite	−1.665	0.048	0.341

Total scores are out of 70 for metacognition, out of 28 for motivation; *average score for each factor on a 1–7 scale.

### Concurrent validity

Table 3 shows the correlation matrix. Short Form SRL-SP motivational scores were significantly correlated with GSES (*r* = 0.48), MCS-24 (*r = 0*.25), and the AOF subscale of action control (*r* = 0.26). Short Form SRL-SP metacognitive scores were significantly correlated with GSES (*r* = 0.41) and MCS-24 (*r* = 0.40) scores.

**Table 3:** Spearman rho correlations between age, the short form SRL-SP subscales, and measures of metacognitive self, general self-efficacy, and action-control subscales.

Variable	1	2	3	4	5	6	7
15. Age	—						
16. MCS-24	0.15*(0.02)	—					
17. GSES	0.07	0.28***(0.08)	—				
18. Metacognitive SRL-SP	0.11	0.40***	0.41***(0.17)	—			
19. Motivational SRL-SP	−0.02	0.25***	0.48***	0.55***(0.30)	—		
20. AOF	0.05	−0.16*	0.30***	−0.06	0.26***(0.06)	—	
21. AOD	0.04	−0.01	0.33***	0.03	0.12	0.31***(0.10)	—
21. AOP	0.14	0.13	0.02	0.10	0.08	−0.07	0.31***(0.10)

* p < 0.05; ** p < 0.01; *** p < 0.001; Numbers in parentheses along the diagonal represent R^2^ values for discriminant validity analyses; AOF = Failure-related action orientation vs. preoccupation subscale; MCS-24 = Metacognitive self-scale; GSES = General self-efficacy scale; AOD = Decision-related action orientation vs. hesitation subscale; AOP = Performance-related action orientation vs volatility subscale.

## Discussion

The study examined how the Polish version of the Short Form SRL-SP performed according to multiple facets of validity. The Polish short form demonstrated better factorial validity for a two-factor model, comprising a motivational and metacognitive subscale, than a global factor. Based on our sample, the Polish Short Form better lends itself to assessment with two factors, and explains greater variance and fit in the modeled data, which is consistent with what Wilson et al. (2019) also reported in the English version in a sample of competitive North American athletes. The two-subscale solution also offers nuance between the two dimensions of SRL, which is a merit compared to the single scale. The one caveat is that the fit indices in the Polish sample fell just short of established criteria for determining acceptable fit, meaning we would advocate some caution in advocating it as an assessment instrument, especially the global factor. As is evident in this investigation, and indicated by Wilson et al., it was never the intention for the Short Form to be advanced solely as an assessment instrument, and thus, the fit indices might be sufficient when considering other merits of this survey. The internal consistency reliability of the Polish Short Form was strong (0.85 for metacognitive, 0.77 for motivational) and in line with the high values for the English version (0.87 for metacognitive, 0.73 for motivational) reported by Wilson and colleagues.

There was strong evidence of group discrimination attesting to the criterion validity of the Polish Short Form SRL-SP. Scores from the metacognitive and the motivational scales differed significantly between each and every competitive sport level, with the only exception for the two least skilled groups on metacognitive scores. The ANOVA analyses indicated that the omnibus group differences were of a medium effect size for motivational scores, and a small effect (just short of medium) for metacognitive scores. Effect sizes from the *post hoc* tests offered evidence of the experts’ advantage. For instance, the international-elite group showed large and medium effects in comparisons to the amateur and regional groups, respectively. The international-elite group also reported small effect size advantages on both subscales over the national group. Moreover, there were generally small to medium effect size differences between all groups across the competitive group simplex. These results are arguably the strongest to date in terms of skill group discrimination (criterion validity) using SRL surveys for several reasons. First, most studies have used two (e.g., Toering et al., 2009, 2012b; Jonker et al., 2010; Reverberi et al., 2021) or three groups (e.g., Bartulovic et al., 2017) for comparative purposes, and four escalating groups provides a more rigorous test (Tedesqui et al., 2018). Secondly, the present trends did not show non-significant anomalies between the least and most skilled groups as was the case in recent work with the full version of the SRL-SP (McCardle et al., 2018). Overall, the effect sizes were somewhat larger and more consistent across more group comparisons than prior research using SRL surveys with three or more skill groups. These findings are a robust example of complete correspondence, with statistical significance at each escalating skill step, suggesting that both metacognitive and motivational SRL competencies contribute to superior sport performance among Polish athletes. The findings add to an evidentiary line which shows significant associations between self-reported SRL in sport practice and markers of sport expertise (e.g., Toering et al., 2009; Wilson et al., 2021), including specifically with the Short Form SRL-SP (Wilson et al., 2019).

Regarding concurrent validity, our results supported the expectation that both motivational and metacognitive subscales would be positively and substantially correlated with general self-efficacy. The strongest correlations, of medium effect size (Ellis, 2010), were between the Short Form SRL-SP subscales scores and GSES scores. The correlation between GSES and the motivational subscale was higher (0.48, nearing a large effect) than the correlation with the metacognitive subscale, which is intuitive considering the motivational subscale shares more content with the GSES around capacity beliefs, confidence, and coping competencies. Theoretically, individuals who believe that they are more capable of performing domain behaviors are more motivated and more likely to be interested in the task (Blecharz et al., 2014). Self-efficacy and capacity beliefs also motivate decisions to initiate an action, the amount of effort athletes will invest, and how long they will persist in behaviors when difficulties appear, all aspects of motivated self-regulation (Rogowska et al., 2022). The correlation between GSES and the metacognitive subscale is interpretable in that more efficacious athletes choose more adaptive practice strategies, including formulating specific goals, and self-evaluating to make attributions for learning (Cleary and Zimmermann, 2001).

A medium-sized correlation was found between the Short Form SRL-SP metacognitive subscale score and metacognitive self, that is, athletes’ report of self-awareness of their self-regulatory biases (Kleka et al., 2019). Biases are personalized tendencies of thinking (Brycz and Karasiewicz, 2011) that are influential in self-regulation of practice. For example, some people have metacognitive biases that lead them to underestimate the time required to achieve a goal or accomplish a task, or to overestimate probability of one’s future success (Kleka et al., 2019), whereas other biases include tendencies to focus on factors facilitating goal pursuit while allocating away from obstacles or evaluation of mistakes (Buehler et al., 1994). A strong metacognitive self, as indicated by MCS-24 scores, indicates individuals have enhanced cognitive capacity for metacognitive skills in learning about oneself (Kleka et al., 2019), thus the medium-sized correlation is evidence of concurrent validity. There was a small-sized correlation between MCS-24 scores and the motivational subscale, which is intuitive, seeing that Kleka et al. (2019) described how a strong meta-cognitive self was associated with a strong motivation to learn about oneself.

In regard to action vs. state orientation subscales, there was little evidence of concurrent validity with the Short Form SRL-SP subscales. Only one relationship provided significant, the small-sized correlation between the AOF subscale (failure-related action orientation vs. preoccupation) and the motivational subscale. Thus, when athletes indicated a greater capability to refocus following failure/disappointment and to dismiss/disrupt bothersome cognitions, they also reported greater use of self-motivation processes to recruit personal effort and cope with difficulties when tasks become hard. This significant correlation is intuitive because action-control orientation becomes increasingly valuable when individuals confront demands and require resilience during goal-oriented tasks (GrÖpel et al., 2014), as would be the case when practice becomes unpleasant and athletes are faced with inhibitory control over temptations to quit hard, deliberate practice (Tedesqui and Young, 2015). One explanation for why this correlation is not stronger may be that survey-based methods have limits in effectively priming respondents to consider the essence of challenge, demands, or threats during a goal-oriented task. This has been noted by action-control researchers, for example, who have used ego-depleting exertion tasks and standardized vigilance tasks to better understand the effects of an individual’s tendencies for action orientation (e.g., GrÖpel et al., 2014). The remaining non-significant correlations between action-control scales and the Short Form SRL-SP subscales might also be explained similarly, that survey methods may have their limits in establishing concurrent validity, when the theoretical construct being assessed might depend on *in situ* priming of significant challenges, or hardship, or ruminating bothers, for individuals to manifest self-regulated decision-making (and not hesitation) and continued on-task regulation (rather than volatility).

Our large, mixed-sport sample may also have been unsuited to show the concurrent validity we expected. For example, Beckmann and Kazén (1994) described how associations between action-control orientation scores and key phenomena are complex and can vary substantially by sport type. Whether a sport is “impulsive,” “controlled,” whether it is “feedback” (which would require more dynamic and constant self-regulation) or “flow”-based (see Beckmann and Kazén, 1994), could moderate correlations between the Short Form SRL-SP measures and action-control scale scores. For instance, athletes with high AOF perform better in “feedback” type sports disciplines, whereas the opposite trend is observed for “impulsive” sport athletes (i.e., long jump, high jump, javelin; Beckmann and Kazén, 1994). Although we recruited intensively and pragmatically required a mixed-sport sample to satisfy sample size requirements for the various statistical tests, we could not pursue any questions about specific sport types, which is a limitation, and which could be an important area of future research.”

### Limitations and future research

Although a strength of this investigation was the use of four escalating skill, allowing for more discriminating inferences on the role of SRL processes toward skill group membership, a limitation was the absence of cross-validation of such groups using secondary performance measures or coach ratings. The current study did not specifically assess SRL measures, or the other concurrent measures for that matter, in relation to indices of practice. Indeed, the current study did not examine how differences in amounts of sport-specific practice contribute to sports-level group status, which may be viewed as a limitation. To most fully validate the role of SRL measures in enhancing practice among Polish athletes, studies should be conducted to examine the relation between report on the Polish Short Form SRL-SP and amounts of practice and particularly in relation to indices of quality/purposeful sport practice. Additionally, future researchers could consider examining how Short Form SRL-SP measures mediate the relationship between sport-specific practice and sports-level group status. Finally, there are limits to cross-sectional analyses such as those employed in the current investigation; thus, longitudinal studies are needed to determine causality and effectiveness of SRL processes on the acquisition of better sport performances.

Given how mixed results for concurrent and psychometric validity were contextualized by the strong evidence for criterion validity, it seems reasonable for future research to inquire about the practical validity of the Polish Short Form SRL-SP. Practical validity refers to the consideration of how research findings/products, in our case the Polish Short Form SRL-SP, are informed by the perspectives of practitioners, located within the narratives of applied practice, and examined instrumentally by those in practice. Young et al. (in press) made the case that such practical validity—evidence for the merits of how an SRL survey can be used as a tool for development, self-learning, and as a catalyst for discussions between athletes and practitioners (e.g., coaches, sport psychology consultants), is a future area of understanding. We agree that this would be valuable for the Polish Short Form SRL-SP, especially given Wilson et al. (2019) purposeful inclusion of consultants’ perspectives in the initial vetting of the English short form to ensure that it could be subjected to examination in practical settings. Further, it is worth examining how our findings extend to younger samples since more evidence is needed to better understand if SRL is a sport-specific skill that can be taught or an individual disposition discriminating among athletes at different competitive levels.

## Conclusion

Altogether, the multiple forms of validation used in our study provided promising results for the use of a Short Form SRL-SP version among Polish athletes. The results provided evidence for very good criterion validity, showing that scores on the survey resulted in stronger and more consistent skill group differences, corresponding completely across all escalating skill groups, than all prior research works using SRL surveys. Our findings suggest that generally greater engagement in metacognitive and motivational processes of SRL distinguishes the most elite group from lesser-skilled groups. There are also differences between amateur-, regional- and national-level athletes. Evidence toward concurrent validity seemed mixed. On the one hand, we found medium-sized correlations with self-efficacy, and medium and small-sized correlations with metacognitive self, yet the concurrent validity in relation to action control was less robust, with only a small-sized correlation between the failure-related action orientation vs. preoccupation subscale and the motivational SRL-SP subscale.

## Data availability statement

The raw data supporting the conclusions of this article will be made available by the authors, without undue reservation.

## Ethics statement

The studies involving human participants were reviewed and approved by The University of Physical Education in Krakow (grant 219/BS/INS/2019). The patients/participants provided their written informed consent to participate in this study.

## Author contributions

MS was responsible for the conceptualization of the research, the research design, participant recruitment, data collection and analysis, statistical analysis, and was a primary contributor to the writing of the first manuscript draft. SW contributed to the data analysis, statistical analysis, and the writing of the manuscript. JB contributed to the research design, was responsible for participant recruitment, and applied his expertise in sport psychology across the manuscript with a focus on the application to sport practice. BY contributed to the data analysis, statistical analysis, and the writing of the manuscript, and edited the final manuscript. All authors contributed to the article and approved the submitted version.

## Funding

This study was financed by The University of Physical Education in Krakow (grant 219/BS/INS/2019). Title of the project: “Translation and adaptation of *A Short Form of the Self-Regulated Learning for Sport Practice Survey SRL-SP SF.”*

## Conflict of interest

The authors declare that the research was conducted in the absence of any commercial or financial relationships that could be construed as a potential conflict of interest.

## Publisher’s note

All claims expressed in this article are solely those of the authors and do not necessarily represent those of their affiliated organizations, or those of the publisher, the editors and the reviewers. Any product that may be evaluated in this article, or claim that may be made by its manufacturer, is not guaranteed or endorsed by the publisher.

## Footnotes

^1^Obliminal rotation was used due to the strong correlation between factors 1 and 2 (*r* = 0.68).

^2^Recruitment was disrupted by COVID restrictions, which limited access to participants and the time they were available. As such, it was not always possible to use all three measures of concurrent validity.
