# Supplementary material for: Examining the validity of the polish short form version of the self-regulated learning—sport practice survey among competitive athletes
Source: Front Psychol. 2023 Feb 2;14:1132608. doi: 10.3389/fpsyg.2023.1132608 (PMC9931730; doi:10.3389/fpsyg.2023.1132608)
Supplement: Supplementary file 1 [file Table_1.docx]

**Appendix 1**

**SRL-SP Skrócona forma (Polish Short Form SRL-SP)**

***Short Form SRL-SP (corresponding English version in italics)***

Wprowadzenie:

Poniższe stwierdzenia dotyczą tego co robisz przed, w trakcie oraz po zadaniach praktycznych.

Zadania praktyczne to jest to, co robisz podczas treningu, gdy starasz się poprawić swoje wykonanie: pracowanie nad umiejętnościami, wykonywanie ćwiczeń, żeby poprawić technikę, prowadzenie strategicznych zagrań z kolegami z drużyny, realizowanie zestawów interwałowych itp.

Proszę przeczytaj te stwierdzenia i oceń, jak często postępujesz zgodnie z opisem.

Nie ma prawidłowych odpowiedzi – opisz siebie takim, jakim jesteś, a nie takim, jakim chcesz być, lub myślisz, że powinieneś być.

*Preface:*

*The following statements are about what you do before, during, and after practice tasks. Practice tasks are what you do during training to try to improve your performance: working on skills, practicing drills to improve technique, running strategic plays with your teammates, completing interval sets, etc. Please read the statements and rate how often you act the way that is described. There are no right answers - please describe yourself as you are, not how you want to be or think you should be.*

| **Prawie nigdy** | |  |  | **Czasami** |  |  | **Prawie zawsze** |
| --- | --- | --- | --- | --- | --- | --- | --- |
| **1** | **2** | | **3** | **4** | **5** | **6** | **7** |

| ***Almost Never*** | |  |  | ***Sometimes*** |  |  | ***Almost Always*** |
| --- | --- | --- | --- | --- | --- | --- | --- |
| ***1*** | ***2*** | | ***3*** | ***4*** | ***5*** | ***6*** | ***7*** |

1. Zanim zacznę robić zadanie na treningu, staram się zrozumieć jego cel.

*I try to understand the goal of a practice task before I do it.* 1 2 3 4 5 6 7

1. Jestem w pełni świadoma/y swoich celów oraz tego, jak intensywnie chcę pracować

na treningu.

*I consciously have goals in mind for how hard I want to work at practice.*

1 2 3 4 5 6 7

1. Podczas realizowania zadań na treningu, sprawdzam jak dobrze sobie radzę.

*I check how well I am doing during practice tasks.* 1 2 3 4 5 6 7

1. Zanim zabiorę się za wykonywanie ćwiczeń, dokładnie planuję, co konkretnie

mam robić.

*I clearly plan my course of action before starting practice tasks.*  1 2 3 4 5 6 7

1. Podczas treningu mam pełną świadomość, że moim celem jest poprawienie sposobu

trenowania.

*During practice, I consciously have goals in mind to improve how I train.*

1 2 3 4 5 6 7

1. Myśląc o tym, co robiłam/-em na treningu, zastanawiam się czy mogę coś poprawić.

*I reflect upon my actions at practice to see whether I can improve them.*

1 2 3 4 5 6 7

1. Zanim wykonam zadanie, układam sobie w głowie poszczególne kroki.

*Before I do a practice task, I think through the steps in my mind.* 1 2 3 4 5 6 7

1. Myśląc o swoim treningu, zastanawiam się nad moim mocnymi i słabym stronami.

*When thinking about my practice, I reflect about my strengths and weaknesses.*

1 2 3 4 5 6 7

1. Opracowuję plan rozwiązywania trudności w praktyce.

*I develop a plan for resolving difficulties at practice.* 1 2 3 4 5 6 7

1. Po zakończeniu ćwiczeń, wracam do nich i oceniam swoje wykonanie

*After finishing, I look back on practice tasks to evaluate my performance.*

1 2 3 4 5 6 7

**Zaznacz proszę, w jakim stopniu zgadzasz się z poniższymi stwierdzeniami:**

***Please indicate your agreement with each of the following statements:***

| **Zdecydowanie nie zgadzam się** | | **Nie zgadzam się** | **Trochę się**  **nie zgadzam** | **Ani się zgadzam, ani nie zgadzam** | **Trochę się zgadzam** | **Zgadzam się** | **Zdecydowanie zgadzam się** |
| --- | --- | --- | --- | --- | --- | --- | --- |
| **1** | **2** | | **3** | **4** | **5** | **6** | **7** |

| ***Strongly***  ***Disagree*** | | ***Disagree*** | ***Slightly***  ***Disagree*** | ***Neither Agree***  ***or Disagree*** | ***Slightly***  ***Agree*** | ***Agree*** | ***Strongly***  ***Agree*** |
| --- | --- | --- | --- | --- | --- | --- | --- |
| ***1*** | ***2*** | | ***3*** | ***4*** | ***5*** | ***6*** | ***7*** |

1. Nawet jeśli nie lubię jakiegoś zadania na treningu, ciężko pracuję.

*Even when I don't like a task during practice, I work hard.* 1 2 3 4 5 6 7

1. W czasie treningu mogę polegać na moich zdolnościach radzenia sobie

z trudnościami.

*When facing difficulties at practice I can rely on my coping abilities.* 1 2 3 4 5 6 7

1. Jestem pewna/-y, że na treningu potrafię skutecznie radzić sobie z nieoczekiwanymi

zdarzeniami.

*I am confident that I can deal efficiently with unexpected events at practice.*

1 2 3 4 5 6 7

1. Zazwyczaj pracuję ciężko i wytrwale, nawet, gdy zadania treningowe stają się trudne.

*I usually keep working hard even when sport training tasks become difficult.*

1 2 3 4 5 6 7
